# Uncovering middle managers' role in healthcare innovation implementation

**DOI:** 10.1186/1748-5908-7-28

**Published:** 2012-04-03

**Authors:** Sarah A Birken, Shoou-Yih Daniel Lee, Bryan J Weiner

**Affiliations:** 1 Lineberger Comprehensive Cancer Center, University of North Carolina at Chapel Hill 1107-A McGavran-Greenberg Campus Box 7411 Chapel Hill, NC 27599-7411, USA; 2 Department of Health Management and Policy, University of Michigan School of Public Health, 1420 Washington Heights, Ann Arbor, Michigan 48109-2029, USA; 3 Department of Health Policy and Management, University of North Carolina at Chapel Hill, McGavran-Greenberg Hall, Campus Box 7411, Chapel Hill 27599-7411, USA

**Keywords:** Middle managers, Healthcare, Innovation implementation

## Abstract

**Background:**

Middle managers have received little attention in extant health services research, yet they may have a key role in healthcare innovation implementation. The gap between evidence of effective care and practice may be attributed in part to poor healthcare innovation implementation. Investigating middle managers' role in healthcare innovation implementation may reveal an opportunity for improvement. In this paper, we present a theory of middle managers' role in healthcare innovation implementation to fill the gap in the literature and to stimulate research that empirically examines middle managers' influence on innovation implementation in healthcare organizations.

**Discussion:**

Extant healthcare innovation implementation research has primarily focused on the roles of physicians and top managers. Largely overlooked is the role of middle managers. We suggest that middle managers influence healthcare innovation implementation by diffusing information, synthesizing information, mediating between strategy and day-to-day activities, and selling innovation implementation.

**Summary:**

Teamwork designs have become popular in healthcare organizations. Because middle managers oversee these team initiatives, their potential to influence innovation implementation has grown. Future research should investigate middle managers' role in healthcare innovation implementation. Findings may aid top managers in leveraging middle managers' influence to improve the effectiveness of healthcare innovation implementation.

## Background

Middle managers' responsibility in healthcare organizations has grown as teamwork designs have become popular in the industry. Teams of clinicians and administrators are often tasked with implementing healthcare innovations [1]. Middle managers--employees who are supervised by an organization's top managers and who supervise frontline employees [2]--oversee the implementation of these innovations [3]. As middle managers' responsibility in healthcare organizations increases, their potential influence over innovation implementation grows.

Despite their potential influence, middle managers have received little attention in extant healthcare innovation implementation research. A comprehensive understanding of middle managers' role in innovation implementation is important because of the gap between evidence of effective care and practice. One study found that patients received just one-half of recommended preventive care, acute care, and care for chronic conditions; some patients received care that was not recommended and was potentially harmful [4]. Further, disparities in access to care and health outcomes exist among racial and ethnic minorities, low-income individuals, and women: In comparison with whites, racial and ethnic minorities suffer from higher rates of diseases such as cancer, obesity, and AIDS [5]. Despite recent concerns about disparities in cardiovascular care, men continue to receive better treatment for heart disease than women [6]. Research has also found significant variation in measures of healthcare quality such as safety climate and patient experience across practice sites and units [7,8]. Many of these disparities can be attributed to failure of healthcare organizations to provide evidence-based care.

The gap between evidence and practice can be closed only if healthcare organizations begin to adopt evidence-based practices. However, implementing even seemingly simple healthcare innovations has proven to be challenging. The implementation rates of quality improvement (QI) initiatives, for example, are less than 50% [9]. Poor implementation rates may be due to the substantial organizational changes required for the initiatives [9]. Indeed, implementing innovations is demanding of employees and organizations--cognitively, emotionally, physically, and spiritually. When attempting to implement innovations, organizations face challenges such as misaligned incentives, professional barriers, competing priorities, and inertia [10].

In this paper, we present a theory of middle managers' role in healthcare innovation implementation. The theory is premised on the notion that middle managers have the potential to bridge informational gaps that might otherwise impede innovation implementation. Variation in healthcare quality may be related to poor communication of key strategic and clinical information across practice sites and units [7,8]. By bridging informational gaps, middle managers may help to manage the demands associated with innovation implementation, align incentives, transcend professional barriers, and identify priorities to promote innovation implementation.

We use the Health Disparities Collaborative (HDC) to provide practical examples of the constructs in our theory. The Health Resources and Services Administration's Bureau of Primary Health Care, which funds health centers in underserved communities in the United States, developed the HDC with the goals of eliminating health disparities by narrowing the gap between evidence and practice in federally qualified health centers [11]. The objectives of the HDC were to decrease or delay disease complications, reduce the economic burden, and improve access to quality chronic disease care for underserved populations, and to develop infrastructure, leadership, and expertise in health centers. Participating health centers were expected to use the following strategies: forming QI teams; creating a registry of patients with chronic diseases to help track clinical care; attending one national and three regional learning sessions where teams learned about the Chronic Care Model [12] and Plan-Do-Study-Act cycles using the Breakthrough Series process [13]; and engaging in activities to promote the implementation of the HDC including the HDC listserv, web page, virtual classroom, conference calls, and feedback on monthly HDC progress reports from regional coordinators and employees.

We begin the paper by discussing the gap in extant literature on healthcare innovation implementation, which has paid little attention to middle managers' role, focusing instead on the roles of top managers and physicians. This is followed by a brief review of literature that has suggested that middle managers influence innovation implementation in industries other than healthcare. We then describe four ways in which middle managers may contribute to implementation climate, which in turn influences healthcare innovation implementation effectiveness. This is followed by a brief discussion of factors that may influence middle managers' role in healthcare innovation implementation. We conclude by suggesting areas for research on middle managers' role in healthcare innovation implementation and discussing the implications of our theory for practice.

### Scope

In this paper, we present a theory of how middle manager may influence the effectiveness of healthcare innovation implementation. As such, our theory exclusively focuses on the relationship between middle managers' commitment to healthcare innovation implementation and implementation effectiveness. Our theory lies at the organizational level. Although middle managers in healthcare organizations exercise their influence on other organizational members on an individual basis, our theory focuses on the contributions that middle managers make to promote consistent, high-quality innovation use at the organizational level.

## Discussion

### Middle managers are largely overlooked in extant healthcare innovation implementation effectiveness research

An innovation is 'an idea, practice, or object that is perceived as new by an individual or another unit of adoption' [14]. For example, the HDC was an innovation because, although some employees may have engaged in QI initiatives before the HDC, the HDC was a distinct, major initiative that employed strategies that were unfamiliar to health center employees [15]. Implementation is 'the transition period during which targeted organizational members ideally become increasingly skillful, consistent, and committed in their use of an innovation' [16]. For example, the HDC guided health centers in improving chronic disease care by developing infrastructure, leadership, and expertise through continuous learning, change, and improvement. At first, some employees perceived continuous learning, change, and improvement processes as an inefficient use of time, but ultimately, many employees came to rely on these methods to improve infrastructure, leadership, and expertise. Innovation implementation, then, refers to a process in which organizational members become proficient in their use of a new practice. We posit that innovation implementation is a process that middle managers may facilitate by engaging in activities specifically related to innovation implementation, thereby improving implementation effectiveness. Implementation effectiveness refers to the aggregate, organization-level consistency and quality of targeted organizational members' use of an innovation [16]. It is a multidimensional construct that includes reach (appropriateness), dose of innovation delivered (consistency), dose of innovation received (consistency), and level of integration (fidelity) [17]. Our theory focuses specifically on the degree to which an innovation's components are integrated into its practices (fidelity) because it is indicative of an organization's potential to achieve an innovation's intended outcomes [14,18], and we are interested in assessing middle managers' contribution to healthcare organizations' achievement of innovations' intended outcomes. The presence of community linkages, for example, is one indication that the HDC was effectively implemented; linking patients with community-based resources such as senior centers and exercise programs allowed patients to benefit from resources that are not available within many healthcare organizations.

Despite a growing literature on healthcare innovation implementation, middle managers' role has received little attention. Health services researchers have only recently begun to acknowledge the importance of innovation implementation. Previously, it was often assumed that implementation was unproblematic and would proceed as planned once a decision was made to adopt an innovation. Alexander, however, found that, relative to studies of healthcare innovation adoption, little research has assessed innovation implementation [9]. As a result, estimates of healthcare innovation effectiveness may be misleading. Health services researchers increasingly recognize that, until an innovation is effectively implemented, the results of the innovation itself cannot be assessed.

Despite recent acknowledgement of its importance, the literature on healthcare innovation implementation remains in its infancy. To date, health services researchers have primarily focused on investigating the roles of top managers and physicians in innovation implementation. Early research focused on the role of physicians [19,20], who were identified as key facilitators in the implementation of innovations such as communication skills training, evidence-based practices, and depression QI initiatives [21-23]. Helfrich et al. found that the implementation of new programs in cancer prevention and control research was related to physicians' commitment [24]. Aarons et al. found that organizational support for implementation improved mental health service physicians' attitudes toward implementing evidence-based practices [25]. Flanagan et al. found that training resources such as academic detailing, grand rounds presentations, and clinical meetings increased physicians' acceptance of clinical practice guidelines; in turn, physicians' acceptance of clinical practice guidelines increased implementation effectiveness [26]. In healthcare organizations such as community mental health agencies, researchers have identified physician resistance as a key barrier to implementing QI initiatives [19,27].

Subsequent healthcare innovation implementation research broadened its scope to investigate top managers' role. Levinson et al. noted that implementation of communication skills training was greatly facilitated by top managers' commitment to the innovation [22]. Kimberly and Cook suggested that the effectiveness of innovation implementation in mental health services was likely influenced by top managers' commitment [28]. Empirical research has borne out this notion: Weiner et al. demonstrated that the extent of clinical involvement in hospital QI efforts was positively related to top managers' commitment [29]. Physicians' attitudes toward innovation implementation and, subsequently, the implementation of a depression QI initiative were also positively related to top managers' commitment [21,30]. Likewise, the most commonly cited implementation facilitator cited in Fremont et al.'s study of HIV collaboratives in Veterans Health Administration organizations was top managers' commitment [31]. In their study of the implementation of new programs in cancer prevention and control research, Helfrich et al. also found that implementation policies that promoted a favorable implementation climate were facilitated by top managers' commitment; in turn, this favorable implementation climate increased implementation effectiveness [24]. Proctor et al. provided a particularly good example of the marked focus of healthcare innovation implementation research on top managers [27]. In a study of facilitators and barriers to implementing evidence-based practices, Proctor et al. interviewed mental health agency directors [27]. The directors cited their own support as a primary facilitator.

Middle managers are underrepresented in studies of healthcare innovation implementation. Some authors have emphasized that implementation effectiveness depends on the ability and willingness of individuals to implement them on the frontlines. Yet they leave unspecified how top managers' commitment is translated into action on the frontlines and whose responsibility it is to enable frontline employees to implement innovations [10,19,32]. In their assessment of the roles of employees and project teams in implementing new practices in hospitals, Tucker et al. acknowledged that middle managers supervise these employees and project teams [33]. Freed suggested that lack of proper frontline management (i.e., middle managers) can stymie hospital turnarounds [34]. In their study of middle managers' involvement in healthcare organizations' strategy processes, Floyd and Wooldridge found that middle managers' boundary-spanning position allowed them to influence their superiors in top management as well as frontline employees [35]. Middle managers' influence on frontline employees, they concluded, was positively related to organizational outcomes such as effectiveness, competitive position, efficiency, and financial performance. Similarly, King and Zeithaml found that middle managers in hospitals who were privy to information regarding their organization's competitive advantage were able to convey the information to appropriate employees throughout the organization [36]. Although these papers suggest that middle managers may influence healthcare innovation implementation, none of the papers empirically assessed middle managers' role in healthcare innovation implementation. In this paper, we present a theory of middle managers' role in healthcare innovation implementation to promote such research.

In contrast to research in healthcare, much research in industries other than healthcare has suggested that middle managers influence innovation implementation. This research indicates that middle managers' influence may be positive or negative. On the one hand, middle managers' commitment to innovation implementation has been linked to strategy realization [37], efficiency of operations [35], and implementation speed [38], as well as positive organizational outcomes such as profit growth [39], enhanced competitiveness [38], and overall effectiveness in reaching established goals [35]. On the other hand, there is evidence that middle managers may limit implementation effectiveness by speaking negatively of an innovation, choosing to withhold information about an innovation, stymying the flow of information about an innovation, or preventing frontline employees from engaging in innovation implementation activities. Floyd and Wooldridge, for example, found that middle managers impeded innovation implementation by 'dragging their feet' or pursuing other priorities [37]. Sayer discussed how middle managers revolted against the implementation of an innovation in a public sector organization to maintain their position and power, effectively bringing innovation implementation as originally conceived by top managers to a halt [40]. Huy similarly found that insufficient commitment among middle managers resulted in poor implementation in an information technology firm [41]. Middle manager resistance has also been cited as the primary obstacle to implementing lean practices in manufacturing firms [42]. Ogbonna and Wilkinson found that middle managers in a food retail company who had concerns regarding an innovation implemented the innovation only out of fear of sanction from top managers. The authors warned that this culture of fear may lead to unintended consequences such as demoralization and diminishing performance in other aspects of middle managers' jobs [43]. Indeed, one middle manager who oversaw HDC implementation, discouraged by poor access to financial, human, and training resources, described implementing the HDC as a futile task [44].

Research has helped to identify ways of encouraging middle managers to positively influence innovation implementation in industries other than healthcare. Similar research on middle managers' role in innovation implementation is lacking in healthcare. In this paper, we present a theory of middle managers' role in healthcare innovation implementation with the goals of filling the gap in the literature and stimulating research that empirically examines middle managers' contribution to innovation implementation in healthcare organizations. Such research may help to identify ways of encouraging middle managers to positively influence innovation implementation in healthcare.

### Theory of middle managers' role in healthcare innovation implementation

**Figure 1:**
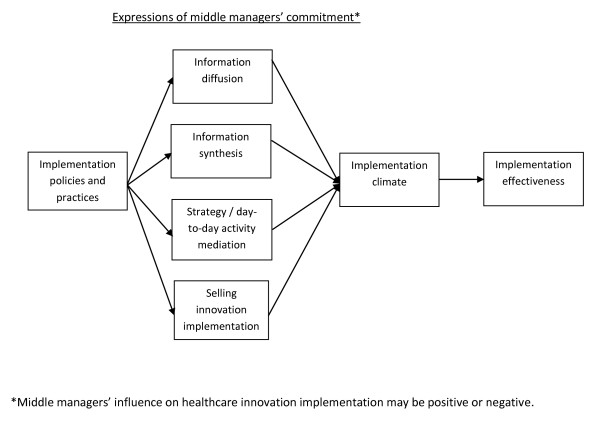
**Middle managers' role in healthcare innovation implementation**.

Figure 1 displays our theory of middle managers' role in healthcare innovation implementation. In colloquial terms, our theory suggests that middle managers give employees information regarding innovation implementation; make it relevant to them; give them the tools necessary to implement innovations; and encourage them to consistently and effectively use those tools. Our theory is premised on the notion that middle managers fill structural holes in healthcare organizations [45]. Middle managers' strategic location between top managers and frontline employees, such as physicians, mid-level providers, medical records staff, and front desk staff, gives them the potential to bridge gaps in information that might otherwise impede innovation implementation. To bridge these gaps, middle managers must commit to innovation implementation. Middle managers' commitment to healthcare innovation implementation is a behavioral manifestation of middle managers' effort and engagement in activities that promote innovation implementation. Specifically, we posit that middle managers express their commitment to healthcare innovation implementation by diffusing and synthesizing information regarding innovation implementation, mediating between strategy and the day-to-day activities required to implement innovations, and selling innovation implementation. These expressions of middle managers' commitment to healthcare innovation implementation include a variety of activities. For example, as we discuss in detail below, selling innovation implementation might involve setting innovation implementation-related norms. Middle managers' influence is likely to be bidirectional: They may disseminate information vertically, from top managers to frontline employees and from frontline employees to top managers, and horizontally, across top managers and across frontline employees.

To effectively implement healthcare innovations, employees require the following types of information: compilation (integrating facts); meta-cognition (interpreting facts); declarative (what to do); procedural (how to do things); and conditional or tacit (when and why to do things) [46,47]. Because middle managers fill structural holes in healthcare organizations, they may bridge the gaps in the information that employees need to effectively implement healthcare innovations by doing the following:

1. Diffusing information. Middle managers disseminate facts, giving employees necessary information about innovation implementation. For example, middle managers who oversaw HDC implementation informed employees that the HDC involved scheduling chronic care follow-up appointments anticipatorily [44].

2. Synthesizing information. Middle managers integrate and interpret facts, making general information about innovation implementation relevant to unique organizations and employees. For some middle managers who oversaw HDC implementation, this involved introducing new systems of patient flow in which front desk staff scheduled chronic care follow-up appointments directly following a visit (anticipatorily) instead of waiting for patients to call for an appointment [44].

3. Mediating between strategy and day-to-day activities. Middle managers identify tasks required for implementing innovations, giving employees the tools necessary to implement innovations. For example, middle managers who oversaw HDC implementation explained how to use appointment systems to schedule chronic care follow-up appointments anticipatorily [44].

4. Selling innovation implementation. Middle managers justify innovation implementation, encouraging employees to consistently and effectively use innovations. For example, HDC middle managers explained that using appointment systems to schedule chronic care follow-up appointments would minimize the need for episodic acute care, improve patient outcomes, and afford greater control over appointment schedules [44].

In turn, we posit, middle managers' commitment to healthcare innovation implementation contributes to an organization's implementation climate--employees' shared perceptions of the extent to which innovation implementation is rewarded, supported, and expected [48]. Middle managers who oversaw HDC implementation, for example, used online tools to find ideas to meet their health centers' needs (an expression of commitment to HDC implementation), contributing to environments that compelled employees to implement the HDC [44]. A strong implementation climate promotes implementation effectiveness--consistent, high-quality innovation use [16]. Health centers in which middle managers contributed to an environment in which employees felt compelled to implement the HDC exhibited more consistent, higher quality HDC use [44]. For example, some middle managers overseeing HDC implementation sought technical assistance from the Health Resources and Services Administration's Bureau of Primary Health Care and relayed the assistance to employees. In so doing, middle managers contributed to an environment in which employees felt compelled to implement the HDC.

Middle managers' ability and willingness to express their commitment to innovation implementation is influenced by implementation policies and practices or IP&Ps--'the plans, practices, structures and strategies that an organization employs...to support innovation [implementation]' [49]. For example, middle managers who underwent performance reviews about HDC-related performance (an IP&P) were more inclined than middle managers who did not undergo such performance reviews to regularly use online tools (an expression of commitment to HDC implementation) [44]. We describe relationships among IP&Ps, middle managers' commitment, implementation climate, and implementation effectiveness below in turn.

### Middle managers diffuse information regarding innovation implementation

Middle managers disseminate facts about innovation implementation, giving employees necessary information about innovation implementation. Middle managers' proximity to both top managers' strategic decisions and frontline employees' day-to-day operations may allow them to relay information regarding innovation implementation to employees. Specifically, staying attuned to top managers' and frontline employees' moods and needs may allow middle managers to provide frontline employees with the information necessary to implement innovations and top managers with feedback regarding innovation implementation status and needs [50,51]. Middle managers overseeing the implementation of the HDC, for example, were tasked with explaining to providers that the HDC would require developing a patient registry. When providers resisted completing the flow sheets used to gather data for the patient registry, middle managers informed top managers of providers' resistance [44].

Scholars in industries other than healthcare have found that middle managers diffuse information regarding innovation implementation. One study found that middle managers in a utility company diffused information regarding an innovation by fielding employees' questions [52]. Another study of organizations outside healthcare found that middle managers used logical presentations of information to attempt to influence top managers [53].

Diffusing information regarding innovation implementation may be particularly important in healthcare organizations. Regulatory pressures and industry standards often make it difficult for employees in healthcare organizations to prioritize tasks. For example, nurses often simultaneously contend with meeting regulatory and professional standards and attending to their organizations' QI initiatives. By disseminating information, middle managers draw employees' attention to innovation implementation.

As information brokers, middle managers contribute to a climate in which innovation implementation is perceived as rewarded, supported, and expected in an organization. By disseminating facts about innovation implementation, middle managers inform employees of an innovation that is expected to be implemented. Ideally, middle managers also disseminate information regarding material or emotional support for innovation implementation and rewards for innovation implementation such as financial incentives or public recognition. As we describe in detail below, middle managers' ability and willingness to diffuse information is influenced by IP&Ps.

### Middle managers synthesize information regarding innovation implementation

Middle managers integrate and interpret facts, making general information about innovation implementation relevant to unique organizations and employees [51,54]. For example, middle managers who oversaw the implementation of the HDC distilled the knowledge that they acquired at national HDC conferences into information that was relevant to their health centers [44]. Synthesizing information may involve activities such as monitoring employees' responses to the information; if middle managers find that an employee has misunderstood the information, they may reinterpret the information in a way that the employee may find more relevant.

Researchers in industries other than healthcare have found that middle managers process information regarding innovation implementation so that it may be useful to employees. Dopson and Stewart, for example, found that middle managers in manufacturing and public sector organizations had a unique vantage that made them privy to issues both within and outside the organization [55]. As a result, middle managers were able to obtain pertinent outside information, which frontline employees could use to gain competitive and strategic advantage for their organization. Similarly, middle managers in textile companies who were privy to information regarding key mechanisms for their organization's competitive advantage were able to contribute to implementation effectiveness by conveying the information to appropriate employees throughout the organization [36]. Mantere also found that middle managers used daily conversations to help frontline employees understand key information regarding innovation implementation [56].

Synthesizing information for employees tasked with innovation implementation may be particularly challenging in healthcare organizations. Hospital teams, for example, are often comprised of hospital employees, such as technicians and nurses, as well as non-staff physicians. Making innovation-related information relevant to this diverse array of employees may be challenging. Because middle managers often span organizational boundaries [57], they may be particularly helpful in bridging informational gaps.

Synthesizing information regarding innovation implementation may contribute to a climate in which innovation implementation is perceived as rewarded, supported, and expected in an organization. For example, middle managers who oversaw the implementation of the HDC synthesized the information that they obtained from online tools for use in their own unique health centers [44]. Interpreting facts about innovation implementation may convey to employees the relevance of the innovation to the specific roles that they are expected to fulfill. Interpreting facts about innovation implementation may also involve explaining to employees the specific ways in which someone in their role would be supported and rewarded for innovation implementation. Again, as we describe in detail below, middle managers' ability and willingness to synthesize information is influenced by IP&Ps.

### Middle managers mediate between strategy and day-to-day activities

Middle managers identify tasks required for implementing innovations, giving employees the tools necessary to implement innovations [58]. In addition to diffusing and synthesizing information, middle managers translate information into concrete tasks that must be carried out to effectively implement innovations. Indeed, Uyterhoeven suggested that middle managers assume the 'bilingual' role of translating strategy into actionable tasks [58]. For example, HDC implementation required health centers to maintain a patient disease registry. To facilitate data collection, middle managers who oversaw HDC implementation introduced new patient flow sheets, developed systems to route patient health records, generated data reports, and troubleshot registry software [44].

Several scholars outside the healthcare industry have found that middle managers' generalist skills allow them the flexibility and adaptability required to translate broad strategy into concrete tasks for implementation [55]. For example, Kanter found that middle managers carried out strategy by giving employees the tools for innovation implementation and practical feedback on their innovation implementation-related performance [59]. Kodama found that middle managers in technology firms transcended divisional barriers to form 'strategic communities' that promoted the implementation of new technologies [60].

Middle managers' ability to translate strategy into actionable tasks may be particularly important in healthcare organizations. Top managers in healthcare organizations, particularly chief executive officers, are often not clinicians [61]. Middle managers in healthcare organizations, on the other hand, are often clinicians such as nurses who have taken on managerial roles. As such, middle managers can translate top managers' strategy, which may seem far removed from clinicians' priorities, into actionable tasks for the clinicians who implement innovations in healthcare organizations.

By mediating between strategy and day-to-day activities, middle managers contribute to a climate in which innovation implementation is perceived as rewarded, supported, and expected in an organization: Middle managers may identify specific activities in which employees are expected to engage to promote an organization's strategy of innovation implementation. In mediating between strategy and day-to-day activities, middle managers also imply that employees will be supported in promoting an organization's strategy of implementing innovations; if employees know what they must do to promote their organization's strategy, they may feel more equipped to do so [47]. Mediating between strategy and day-to-day activities identifies specific activities in which employees may engage to earn associated rewards such as financial incentives. For example, introducing new patient flow sheets conveyed to providers implementing the HDC that data collection was an expected component of patient care. It also suggested that their health centers could support them with the tools necessary to develop and maintain patient registries. And some middle managers who oversaw HDC implementation discussed with providers that using the new patient flow sheets promoted accurate data related to their performance in managing chronic diseases; in turn, accurate performance data would yield fair rewards [44]. Again, as we describe in detail below, middle managers' ability and willingness to mediate between strategy and day-to-day activities is influenced by IP&Ps.

### Middle managers sell innovation implementation

Middle managers justify innovation implementation, encouraging employees to consistently and effectively use innovations [54]. Translating operational information into concrete tasks may be necessary but insufficient to promote effective implementation: Employees must consider execution of those tasks to be a worthy use of their time. By convincing employees that innovation implementation is worthy of their attention, middle managers obtain employees' support for innovation implementation. To counter resistant providers, for example, middle managers who oversaw HDC implementation encouraged them to view the HDC as an efficient way of staying abreast of state-of-the-art clinical practices [44]. Selling innovation implementation may involve setting innovation implementation-related norms. For example, some middle managers who oversaw HDC implementation worked to incorporate data collection into routine health center operations by collecting data themselves and creating systems to encourage others to collect data. Selling innovation implementation may also involve leadership. For example, some middle managers who oversaw HDC implementation viewed maintaining a positive attitude regarding innovation implementation for the benefit of the employees whom they supervised as central to their role in innovation implementation.

Several researchers in industries other than healthcare have found that middle managers champion innovation implementation, encouraging employees to implement innovations. Rouleau (2005) found that middle managers in a Canadian clothing manufacturing organization helped employees to appreciate the rationale underlying organizational changes, thereby promoting innovation implementation [62]. Similarly, Ogbonna and Wilkinson found that middle managers in a food retail company considered central to their role encouraging employees to implement an innovation [43]. Another study found that middle managers in a European professional service influenced both the opinions and activities of frontline employees and their supervisors through daily conversations, subsequently increasing implementation effectiveness [56].

Selling innovation implementation may be particularly important in healthcare organizations. As noted above, clinical and administrative staff often contends with meeting regulatory and professional standards and attending to their organization's service and QI initiatives. By conveying the importance of implementing a particular innovation, middle managers encourage employees to prioritize innovation implementation.

By selling innovation implementation, middle managers contribute to a climate in which innovation implementation is perceived as rewarded, supported, and expected in an organization. Selling innovation implementation suggests that middle managers consider innovation implementation to be worthy of their own attention and, therefore, other employees' attention; that is, innovation implementation is expected of employees. Many middle managers overseeing HDC implementation, for example, viewed themselves as role models; they believed that engaging in HDC-related activities encouraged employees to do so as well [44]. Suggesting that innovation implementation is worthy of middle managers' attention implies that employees will be supported in implementing innovations: If middle managers have the resources necessary to implement innovations, ostensibly, so will other employees. Middle managers are likely to know how employees can successfully fulfill their roles. When middle managers sell innovation implementation, employees may believe that engaging in activities to promote innovation implementation will confer the rewards associated with successfully fulfilling their implementation-related roles. For example, some middle managers reported that being publicly recognized for their HDC-related efforts encouraged employees to engage in HDC-related activities. And when employees saw that some middle managers were discouraged by lack of support from top managers for HDC implementation, their efforts flagged [44]. Again, as we describe in detail below middle managers' ability and willingness to sell innovation implementation is influenced by IP&Ps.

### The influence of implementation policies and practices on middle managers' role in healthcare innovation implementation

We posit that whether or not middle managers influence healthcare innovation implementation and whether their influence is positive or negative is influenced by IP&Ps--'the plans, practices, structures and strategies that an organization employs...to support innovation [implementation]' [49]. The theory of perceived organizational support, a social exchange interpretation of commitment, provides a framework for understanding how IP&Ps may increase middle managers' ability and willingness to express their commitment to innovation implementation [63]. The theory's premise is that employees such as middle managers anthropomorphize their organizations, attributing the actions of organizational agents (e.g., top managers) to the organization's intent [64]. Organizational agents' actions are often responses to the organization's legal, financial, and moral responsibilities, and its policies, practices, and culture, but middle managers who anthropomorphize their organizations view organizational agents' actions as an indicator of the extent to which their organization favors an initiative such as innovation implementation. For example, incentives (an IP&P) were sometimes offered to middle managers whose teams effectively implemented the HDC. Middle managers may have viewed the incentives as an indication that their organization valued HDC implementation [44]. Middle managers may develop affective attachment to innovation implementation because they believe that IP&Ps are a sign that their organization cares about the innovation. As another example, performance reviews about HDC-related performance (an IP&P) signaled to middle managers that the HDC was important to their health centers [44]. This may cause middle managers to commit to innovation implementation, contributing to a climate in which innovation implementation is viewed as rewarded, supported, and expected.

### Pursuing a clearer understanding of middle managers' role in healthcare innovation implementation

Whether middle managers influence healthcare innovation implementation, the ways in which they influence healthcare innovation implementation, and whether their influence is positive or negative warrants empirical research. Such research will fill a gap in extant health services research and will identify ways of encouraging middle managers to positively influence innovation implementation. Middle managers' role may be particularly important for healthcare innovation implementation because they can encourage employees to prioritize innovation implementation amidst many competing demands; transcend complex staffing and operations to provide relevant information regarding innovation to employees; leverage their position over project teams to promote innovation implementation; and translate organizational strategy into clinically relevant terms. Indeed, middle managers have a poorly understood yet potentially critical role in healthcare innovation implementation.

### Summary

The role of top managers and physicians has garnered more attention than the role of middle managers in the small but growing research literature on healthcare innovation implementation--the transition period when employees become adept at using a new practice. Extant theory and research point to physician buy-in and top managers' commitment as key determinants of implementation effectiveness--the consistency and quality of targeted organizational members' use of an innovation [16]. Less theorized or studied is middle managers' role. Teamwork designs have become popular in healthcare organizations [1]. Because middle managers oversee these team initiatives, their influence over innovation implementation has grown [3]. The omission of middle managers from research on healthcare innovation implementation is particularly problematic because middle managers in healthcare organizations are unique. Many middle managers are promoted based on clinical skills but may lack the skills to run a department or service area [65]. In addition, middle managers' role in healthcare innovation implementation may differ from the role of middle managers in other industries because middle managers in healthcare organizations often assume their management role in addition to clinical responsibilities. Understanding middle managers' role in innovation implementation is critical for improving implementation effectiveness in healthcare organizations. Future research should fill the current gap in extant health services research by investigating middle managers' role in healthcare innovation implementation. Ideally, this research will identify ways of encouraging middle managers to positively influence healthcare innovation implementation.

### Future research on middle managers' role in healthcare innovation implementation

Future healthcare innovation implementation research should address the following questions: Do middle managers influence healthcare innovation implementation, and under what conditions? Is middle managers' influence on healthcare innovation implementation positive or negative, and under what conditions? What are the factors that influence middle managers' commitment to innovation implementation? In what ways do middle managers influence healthcare innovation implementation? Pursuing each of these questions may be facilitated by using the theory of middle managers' role in healthcare innovation implementation presented in this paper.

An initial study of middle managers' role in healthcare innovation implementation would ideally use empirical data to assess whether middle managers influence implementation effectiveness and, if so, whether their influence is positive or negative. Such a study might address this question by assessing the relationship between middle managers' commitment to innovation implementation--that is, behavioral manifestations of middle managers' effort and engagement in activities that promote innovation implementation--and implementation effectiveness. To ensure that the question is adequately addressed, the study must include middle managers with varying degrees of commitment and multiple healthcare organizations implementing a single innovation with varying degrees of effectiveness. This would ensure a consistent measure of implementation effectiveness and produce generalizable results.

Assessing the conditions under which middle managers influence healthcare innovation implementation and the conditions under which middle managers' influence is positive or negative could begin with studying relationships among IP&Ps, middle managers' commitment, and implementation effectiveness. We posit in our theory of middle managers' role in healthcare innovation implementation that middle managers' ability and willingness to engage in innovation implementation-related roles is influenced by IP&Ps. To investigate this theory, health services researchers could assess whether middle managers' commitment mediates relationships between IP&Ps and implementation effectiveness. Alternatively, IP&Ps may moderate the relationship between middle managers' commitment and implementation effectiveness. Both possibilities should be explored in future studies.

Many other variables, such as organizational size, complexity, formalization, and centralization may also moderate the relationship between middle managers' commitment to healthcare innovation implementation and implementation effectiveness. For example, middle managers' commitment may have a weak or nonexistent influence on implementation effectiveness in small or highly centralized healthcare organizations. In these types of organizations, middle managers may have little autonomy, or their role may be indistinguishable from that of top managers. This may be particularly true in small primary care practices in which a single employee fulfills the roles of both top and middle manager.

If, indeed, middle managers' commitment has a relationship with healthcare innovation implementation effectiveness, a subsequent study could assess the ways in which middle managers influence healthcare innovation implementation. Such a study may address this question by determining whether the roles proposed in our theory of middle managers' role in healthcare innovation implementation mediate the relationship between middle managers' commitment and implementation effectiveness and how these roles influence implementation climate. For example, middle managers may prioritize diffusing information over selling innovation implementation. And some roles may contribute to an environment in which innovation implementation is perceived as expected, supported, and rewarded more than other roles. Perhaps, for example, employees feel most compelled to implement innovations when middle managers sell innovation implementation by role-modeling. This information could help top managers to support middle managers in engaging in their roles as effectively as possible. Ideally, a quantitative study would be paired with qualitative methods to gain deeper knowledge of the ways in which middle managers influence healthcare innovation implementation.

Studies of middle managers' role in healthcare innovation implementation would likely pose several conceptual challenges. For example, defining middle managers may not be straightforward. Borrowing from Noble, we conceptualized middle managers as employees who supervise other employees in the organization and are supervised by top managers [2]. However, middle managers may fill positions one, two, three, or more levels below top managers [66]. Middle managers in healthcare organizations may be even more difficult to define because they often have diverse professional backgrounds. Middle managers who oversaw the implementation of the HDC, for example, were represented by physicians, nurses, social workers, and administrators [44]. Further, middle managers in healthcare organizations often have diverse functions; instead of supervising a consistent set of frontline employees, healthcare organizations' teamwork designs require middle managers to simultaneously oversee several projects. Middle managers in healthcare organizations also tend to occupy a variety of positions. Many middle managers who oversaw HDC implementation, for example, had both administrative and clinical responsibilities [44]. Initial empirical research on middle managers' role in healthcare innovation implementation would ideally adopt a broad definition of middle managers. Subsequent research, however, should consider various definitions of middle managers as moderators of the relationship between middle managers' commitment to healthcare innovation implementation and implementation effectiveness.

For several reasons, studying middle managers' role in healthcare innovation implementation may also be methodologically challenging. For example, as mentioned above, a study of the relationship between middle managers' commitment to innovation implementation and implementation effectiveness would assess middle managers' commitment to a single innovation in multiple healthcare organizations. Finding a single innovation that is simultaneously implemented in multiple healthcare organizations is likely to be challenging. Such a study might benefit from capitalizing on multiple healthcare organizations' implementation of innovations required by an accrediting body.

In addition, researchers must account for the multi-level nature of middle managers' role in innovation implementation. Specifically, our theory suggests that IP&Ps (organization-level constructs) influence middle managers' ability and willingness to express their commitment to innovation implementation (an individual-level behavior). By diffusing information, synthesizing information, mediating between strategy and day-to-day activities, and selling innovation implementation, middle managers contribute to a climate in which innovation implementation is rewarded, supported, and expected (an organization-level construct). This implementation climate promotes consistent, high-quality innovation use (an organization-level construct; see Figure 1). Researchers must carefully address these multi-level issues to promote clear understanding of middle managers' role in healthcare innovation implementation.

### Implications for practice

The theory of middle managers' role in healthcare innovation implementation described in this paper suggests that top managers in healthcare organizations may neglect a potentially important determinant of implementation effectiveness. Leveraging middle managers' role may have the potential to increase implementation effectiveness in healthcare. Specifically, the theory of middle managers' role in healthcare innovation implementation suggests that middle managers may influence healthcare innovation implementation in the following ways: diffusing information regarding innovation implementation; synthesizing information regarding innovation implementation; mediating between strategy and the day-to-day activities required to implement innovations; and selling innovation implementation. Top managers in healthcare organizations may promote implementation effectiveness by encouraging middle managers to fulfill these roles. Specifically, top managers could promote the fulfillment of these roles by: Ensuring that middle managers are privy to strategy related to innovation implementation; without information regarding strategy, middle managers would lack the knowledge required to educate, support, and encourage employees to implement innovations; giving middle managers the freedom to educate, support, and encourage employees; ensuring that middle managers have access to resources necessary to translate strategy into day-to-day activities; for example, if top managers envision information systems to be a key strategy for implementing an innovation such as the HDC, then middle managers will require information systems training to better support employees' use of the technology; and expressing their own commitment to innovation implementation; doing so would convey to employees consistent commitment to innovation implementation throughout the organization.

## Competing interests

The authors declare that they have no competing interests.

## Authors' contributions

All authors made significant contributions to formulation of the theory. SB drafted and SYDL and BW critically revised the manuscript for important intellectual content. All authors gave final approval of the version of the manuscript submitted for publication.
